# Transcatheter Aortic Valve Replacement in Special Populations

**DOI:** 10.31083/j.rcm2402049

**Published:** 2023-02-06

**Authors:** Khansa Ahmad, Amgad Mentias, Hafiz Imran, Ayman Elbadawi, Omar Hyder, Paul Gordon, Barry Sharaf, Marwan Saad

**Affiliations:** ^1^Department of Medicine, Division of Cardiology, Alpert Medical School of Brown University, Providence, RI 02903, USA; ^2^Lifespan Cardiovascular Institute, Providence, RI 02903, USA; ^3^Cardiovascular Medicine, Cleveland Clinic, Cleveland, OH 44195, USA; ^4^Cardiovascular Medicine, Baylor College of Medicine, Houston, TX 77030, USA

**Keywords:** aortic valve replacement, transcatheter aortic valve replacement, AVR, bioprosthesis, special population

## Abstract

Since its food and drug administration (FDA) approval in 2011, transcatheter aortic valve replacement (TAVR) has revolutionized the highly prevalent disease of aortic stenosis. In this review, we present a comprehensive overview of the data and considerations for utilization of TAVR in special populations who were either excluded from or not adequately represented in the seminal TAVR trials, due to high-risk valvular and/or systemic factors. These include nonagenarians, patients with renal dysfunction, chronic thrombocytopenia, bicuspid aortic valve, rheumatic valve disease, patients with failed aortic valve bioprosthesis requiring valve-in-valve intervention and patients with mixed aortic valve disease. In short, TAVR is a feasible therapeutic strategy in high-risk and special populations with mortality benefit and improvement in quality of life. Randomized controlled trials in high-risk populations are recommended to confirm results from observational studies.

## 1. Introduction 

Aortic stenosis (AS) is the second most common valvular lesion in the United States, following mitral regurgitation, with a prevalence of about 12.4% in the elderly [1]. In patients above 75 years of age, 2%–4% have severe AS [1, 2, 3]. By the time patients present with severe AS, comorbidities acquired with aging render many of them high risk for surgical aortic valve replacement (SAVR). This created a need for a minimally invasive approach for aortic valve replacement (AVR). In 1990, a patent for transcatheter aortic valve replacement (TAVR) was filed, and granted in 1995 [4]. Subsequent research and development led to the Placement of Aortic Transcatheter Valve (PARTNER) trials from 2010 to 2019 as well as the trials for self-expanding bioprosthesis [5, 6, 7, 8]. These trials established the benefit and efficacy of TAVR leading to the approval of Edwards Sapien valve by the food and drug administration (FDA) in 2011, for high-risk patients with severe AS who were not eligible for SAVR. Subsequently, the valve was approved as an alternative to surgery in high-risk (2012), followed by intermediate (2016), and low-risk patients (2019) with severe AS.

In this paper, we review the safety and efficacy of TAVR in special categories of patients who were minimally represented in or totally excluded from the pivotal TAVR trials due to systemic or valvular features that impart elevated risk for poor outcomes.

## 2. Systemic and Patient-Related Factors 

### 2.1 Renal Dysfunction

Patients with significant renal dysfunction with serum creatinine >3 mg/dL and patients with end-stage renal disease (ESRD) on hemodialysis (HD) were excluded from the seminal TAVR trials [5, 8]. This was based on the finding of worse long-term outcomes after SAVR in patients with renal dysfunction and the possibility of contrast-induced nephropathy, microthromboembolic showers, and reduced cardiac output during rapid pacing for valve deployment, worsening the preexisting renal dysfunction [9, 10]. In a study from the Society of Thoracic Surgeons /American College of Cardiology Transcatheter Valve Therapies (STS/ACC TVT) registry including a total of 72,631 patients with severe AS who underwent TAVR, patients with ESRD (n = 3053, 4.2%) were associated with increased risk of in-hospital mortality and major bleeding compared to those who were not on dialysis [11].

Few studies have compared outcomes with TAVR, SAVR, and conservative management in patients with ESRD. In a large observational study from the US, data from the National Medicare Provider and Analysis Review (MEDPAR) Part A files were analyzed to assess outcomes of patients with ESRD on HD who underwent TAVR versus SAVR and showed improved short- to mid-term outcomes after TAVR as compared to SAVR (4.6% versus 12.8%, *p *< 0.01) [12]. Furthermore, these patients had improved outcomes after TAVR as compared to conservative management (adjusted hazard ratio [HR] 0.53 [95% CI, 0.47–0.60], *p <* 0.001). Patients who underwent TAVR or SAVR were found to have a significant reduction in rates of heart failure admissions after the valve replacement for up to 1-year (TAVR incidence rate ratio, 0.55 [95% CI, 0.48–0.62], *p <* 0.001; SAVR incidence rate ratio, 0.76 [95% CI, 0.65–0.88], *p <* 0.001) [13].

A second retrospective observational study from European centers in France and Belgium showed that moderate and severe chronic kidney disease (CKD) was associated with a significant increase in all-cause (adjusted HR [95% CI] =1.36 [1.08–1.71], *p* = 0.009) and cardiovascular mortality (adjusted HR [95% CI] = 1.39 [1.03–1.88], *p *= 0.031) [14]. However, propensity-matched analysis showed that aortic valve replacement (SAVR or TAVR) led to a significant improvement in all-cause and cardiovascular mortality at 5 years, irrespective of the CKD stage, relative to conservative management (Cox time varying covariate *p *< 0.001 for mild and moderate CKD, and Cox time varying covariate *p *= 0.0016 for severe CKD) [14]. Subgroup analysis of the PARTNER 1, 2, and 3 trials for patients with CKD revealed that the estimated glomerular filtration rate (eGFR) was improved or remained largely unchanged after TAVR procedure [15].

These findings would indicate that, despite being a higher risk cohort, patients with renal dysfunction may still derive significant benefit from undergoing valve replacement and warrant a shared decision-making to address risks and benefits. In patients who are not yet dialysis dependent, judicious use of contrast during TAVR is critical.

### 2.2 Thrombocytopenia

Due to the need for large bore access, there is a risk of major bleeding associated with TAVR procedure, found to be 10.2% (± 3.5) at 30 days and 16.0% (± 0.9) at 1 year, in an analysis from the Surgical Replacement and Transcatheter Aortic Valve Implantation (SURTAVI), PARTNER 2 cohort and the US Coevolve high-risk study [16]. Patients with chronic thrombocytopenia (platelet count <50,000 cells/${\mathrm{mm}}^{3}$) were excluded from the pivotal TAVR trials [5]. The literature is robust when it comes to post-procedural thrombocytopenia with TAVR and the associated risk of complications such as post-procedural packed red blood cells and platelet transfusion, major bleeding, vascular complications, and long critical care unit length of stay [17]. However, there are fewer studies examining outcomes of patients with chronic thrombocytopenia undergoing TAVR. Studies have conflicting results with regards to post-TAVR short- and long-term mortality associated with chronic thrombocytopenia. A propensity-matched cohort from the National Inpatient Sample (NIS) from 2012–2015 showed increased in-hospital mortality in patients with chronic thrombocytopenia (6.0% versus 3.3%, *p* value 0.007) [18]. However, another propensity-matched analysis of the NIS up to 2014 showed no difference in in-hospital mortality (4.5% versus 5.7%; adjusted odds ratio [adjOR] 0.79; 95% CI 0.57–1.09; *p* = 0.16) [19]. All studies indicate a higher risk of post procedural vascular complications, post-procedural blood transfusions, acute kidney injury (AKI), cardiogenic shock, cardiac tamponade, and hemopericardium. There is also evidence that supports higher cost and resource utilization in patients undergoing TAVR in the setting of chronic thrombocytopenia, particularly in patients who develop post-procedural complications [18]. In almost all studies, the authors found that thrombocytopenia was associated with the presence of multiple comorbidities and proposed that it might be a marker of frailty. In general, patients with thrombocytopenia should undergo rigorous screening prior to the procedure. Furthermore, precautions to avoid access site complications such as utilizing ultrasound-guided access and closure devices by experienced personnel may help mitigate post-procedural complications and resultant morbidity and resource utilization. Further prospective analyses to evaluate outcomes, complications, risk stratification, and precautionary measures are warranted to develop an optimal strategy when it comes to treating patients with chronic thrombocytopenia and severe AS.

### 2.3 Nonagenarians 

Aortic stenosis is a disease of age (barring congenital malformations). In the PARTNER-I trial, there were 531 patients above 90 years of age; 329 of them underwent transfemoral and 202 underwent transapical TAVR [5]. This amounted to 50% of the high risk and nonsurgical cohort who were randomized to TAVR versus conservative management. These patients had improvement in their functional status from baseline. Furthermore, patients who had undergone transfemoral TAVR had a slightly higher stroke risk (3.6% versus 2%) but improved 30-day and three-year mortality as compared to transapical TAVR. Subsequent analysis using the Transcatheter Valve Therapy registry has shown similar outcomes between nonagenarians and octogenarian [20]. Utilizing Medicare data, Mentias *et al*. [21] demonstrated a temporal trend of improved outcomes in both patients >90 years (9.8% in 2012 to 4.4% in 2016; *p <* 0.001) and younger (6.4% to 3.5% in 2016; *p *< 0.001). Furthermore, in high-volume centers, there was no difference in mortality between nonagenarians and younger patients (2.2% versus 1.7%; odds ratio: 1.33; 95% CI, 0.97–1.81; *p *= 0.07) [21]. Procedural complications such as AKI, in-hospital stroke, and respiratory complications were associated with worsening 30-day mortality. Operator experience, femoral approach, and a careful evaluation of the patients prior to the procedure can help improve procedural outcomes and reduce complications.

## 3. Valvular Factors 

### 3.1 Bicuspid Aortic Valve

Bicuspid aortic valve (BAV) is the most common congenital anomaly of the valves representing about 25% of patients >80 years referred for AVR [22]. However, this valve morphology was excluded from the pioneering TAVR trials due to concerns about clinical outcomes. High-risk anatomical features of BAV include higher degree and non-uniform distribution of calcification, as well as non-circular annuli; those can predispose to high incidence of periprocedural strokes, perivalvular leaks, and potential risk for annular rupture [23]. Furthermore, BAV is associated with a high incidence of ascending aorta aneurysm. Data from the STS registry and NIS has been analyzed in observational cohorts to assess the short-term outcomes and temporal trends and compare outcomes of SAVR versus TAVR in patients with BAV. Both studies demonstrated similar in-hospital mortality for TAVR versus SAVR for BAV [24, 25]. Elbadawi *et al*. [25] also showed that TAVR had similar in-hospital mortality for tricuspid and bicuspid anatomy (2.9% versus. 3.4%; OR: 0.85; 95% CI: 0.30–2.40; *p* = 0.76). The rates of post-procedural myocardial infarction, bleeding, vascular complications, and discharge to nursing facility were similar between SAVR and TAVR, however TAVR was found to be associated with higher rates of complete heart block and permanent pacemaker insertion [25]. In another study utilizing Medicare database, there was no difference in in-hospital mortality in propensity-score matched cohorts of patients with BAV who underwent TAVR versus SAVR [26]. The study by Makkar *et al*. [24], similarly highlighted that TAVR had similar mortality for bicuspid and tricuspid AS at 30 days (0.9% versus 0.8%; HR, 1.18 [95% CI, 0.68–2.03]; *p* = 0.55) and at 1 year (4.6% versus 6.6%; HR, 0.75 [95% CI, 0.55–1.02]; *p* = 0.06) as well as similar stroke rates. They also demonstrated that procedural complications, post-procedural hemodynamics and, moderate to severe perivalvular leak were not significantly different for the two anatomic variants [24].

### 3.2 Rheumatic Valve Disease

Although there has been a decline in the prevalence of rheumatic heart disease (RHD) in the developing world, it still represents a significant clinical burden of disease in the low-income countries [27]. Pathologically, RHD is associated with fibrotic changes in the aortic valve anatomy, rather than a purely calcified degeneration which represents a unique challenge in terms of appropriate deployment and anchoring of the TAVR bioprosthesis. Therefore, aortic stenosis due to RHD was excluded from the pivotal randomized control trials for TAVR [5, 8]. However, newer iterations of TAVR valves have been designed to improve anchoring and reduce prosthesis migration and paravalvular leak. New valves (e.g., JenaValve) are being examined in patients with predominant aortic regurgitation and can be used in RHD patients with less degree of annular calcification and concerns about anchoring [28]. In an analysis of Medicare patients from 2015 to 2017, Mentias *et al*. [29] showed that there was no difference in 30-day and mid-term mortality in patients with rheumatic AS who underwent SAVR versus TAVR (11.2 versus 7.0 per 100 person-year, HR 1.53, 95% CI 0.84–2.79, *p* = 0.2). Furthermore, TAVR had similar outcomes for patients with rheumatic versus nonrheumatic AS in terms of in-hospital and mid-term mortality, 30-day stroke, heart failure admissions, AKI, and blood transfusion. TAVR was also found to have a favorable intermediate-term outcome in a median follow-up of 19 months, with a lack of need for repeat valve replacement or repair.

### 3.3 Valve-in-Valve

Bioprosthetic valves carry the inherent risk of structural valve deterioration (SVD) within 10–20 years [30]. Historically, the only alternative in patients suffering from SVD was redo SAVR which comes with a higher operative risk compared to a primary valve replacement [31, 32]. Valve-in-Valve (ViV) TAVR not only offers a lower risk alternative for patients afflicted with SVD of a bioprosthetic valve but may also have implications on initial selection of mechanical versus biological prosthesis. Over the past two decades, TAVR has been proven to be a viable option for all degrees of surgical risk leading to an increase in TAVR implantation. Inevitably, ViV TAVR has therefore been pursued, with techniques being refined over time for both prior surgical valves as well as TAVR valves. According to the most recent report from the STS-ACC TVT registry, planned ViV TAVR is increasing, the majority of which are performed for failed surgical bioprosthetic valves (TAVR-in-SAVR), with more than 15,000 cases performed between 2012 and 2019, and only 404 TAVR-in-TAVR cases performed over the same period [33].

A meta-analysis of 23 studies, through July 2020, showed no difference in 30-day and 1-year mortality as well as 30-day stroke risk of ViV TAVR compared with either redo SAVR or primary TAVR [34]. A more recent systematic review and meta-analysis of 9 studies, with a total of 9100 patients, showed lower 30-day mortality associated with ViV TAVR (OR, 0.56; *p <* 0.0001) when compared to redo SAVR, but similar mortality at follow-up (HR, 1.02; *p *= 0.086) [35]. It is important to note, however, that redo SAVR was associated with a lower risk of paravalvular leak (PVL), severe patient prosthesis mismatch, and lower post-procedural aortic valve gradients.

Deployment of ViV transcatheter heart valve (THV) has several important concerns and considerations, including coronary ostial obstruction, patient prosthesis mismatch, elevated postprocedural gradients, high-grade atrioventricular block requiring permanent pacemaker placement, and leaflet thrombosis. One of the current challenges is confirming optimal THV expansion during the procedure, which is critical to achieving adequate blood flow restoration, and reducing PVL, and residual gradients. While preprocedural CT scan assessment is the current standard of care to estimate the nominal THV sizing and the required percentage of oversizing, it may not correlate well with the actual THV expansion which is impacted by many factors including the degree of landing zone calcification, 3D anatomy, etc. Currently, there is no validated measure to assess the actual THV expansion in real time intraprocedurally. A case series from Poland published in 2020 described the use of large field intravascular ultrasound to guide and assess TAVR deployment. This may represent a step toward exploring a valid method of optimizing THV deployment and reducing postprocedural transvalvular gradients and PVL. Further studies are needed to assess optimal measures in this regard [36].

A virtual distance of <3 mm between the coronary ostium and the TAVR valve has been shown to be highly predictive of coronary artery obstruction [37]. Pre-TAVR imaging with CT scan to assess this distance can help in procedural planning such as having a provisional guide catheter and guidewire in the at-risk coronary artery or employing the laceration of valve leaflets technique to prevent coronary obstruction (BASILICA procedure) [38, 39, 40]. Similarly, female gender and small internal diameter of the surgical valve are highly predictive of post-procedural patient prosthesis mismatch and elevated gradients [41]. Patient prosthesis mismatch itself is a marker for adverse outcomes after ViV TAVR. This can be mitigated by employing self-expanding valves and high supra-annular deployment of the bioprosthesis. All these considerations should be included in planning the first bioprosthetic valve procedure for treating aortic stenosis to facilitate the future ViV procedure in patients who may need a second valve in their lifetime. In patients with a small aortic annulus, aortic root enlargement should be considered at the time of first SAVR to allow a larger bioprosthetic valve which subsequently will allow for a larger ViV bioprosthesis, and hence reducing the risk of coronary obstruction and patient-prosthesis mismatch, with the goal to improve long-term outcomes.

### 3.4 Mixed Aortic Valve Disease

Mixed aortic valve disease (MAVD) is defined as presence of both severe AS and moderate to severe aortic regurgitation (AR). This patient population was excluded from the landmark TAVR trials and the guidelines for management of valvular heart disease recommended evaluation of individuals and treatment according to the predominant lesion [5, 6, 7, 8, 42, 43]. The pathophysiology of MAVD is unique as the left ventricle is faced with both an increased afterload due to severe AS and volume overload due to AR. Since the seminal TAVR trials, several observational analyses have demonstrated similar or improved outcomes in patients with MAVD as compared to pure AS, especially in patients who developed post procedural AR. In a single center study of 1133 patients, Chahine *et al*. [44] demonstrated improved survival and lower 3-year mortality rates in patients with MAVD versus pure AS (15.3% versus 20.4%; *p* = 0.02). This effect was driven predominantly by improved survival in patients who developed post TAVR AR. Similar findings were demonstrated in a cohort of 622 patients from Cleveland clinic; although moderate or severe central or paravalvular AR was more common (15.5% versus 6.7%, *p* = 0.004) and device success was less prevalent in MAVD (81% versus 88.9%, *p* = 0.027), the univariable survival was better in patients with MAVD as compared to pure AS (71.3% versus 62.6%; *p* = 0.02) [45]. Grant *et al*. [46] published an analysis utilizing Nationwide Readmissions Database, identifying 100,573 TAVR patients and 3260 patients with MAVD. In this study, in-hospital mortality (2.5% versus 2.6%, *p* = 0.53) and rates of paravalvular leak (1.0% versus 1.3%, *p* = 0.05) were similar in MAVD versus pure AS, MAVD was not a significant predictor of mortality (adjusted odds ratio [adjOR] 1.25, *p* = 0.05), was associated with decreased odds of 30-day (adjOR 0.05, *p *< 0.001) and 90-day readmission (adjOR 0.04, *p *< 0.001) and higher odds of pacemaker implantation (adjOR 1.46, *p *< 0.001) [46].

The improvement in survival in MAVD patients, especially in the cohort with post procedural AR is proposed to be driven by the preexisting remodeling to accommodate AR as compared to patients who face this challenge de novo post procedurally, i.e., patients with pure AS. Further randomized controlled trials are needed to confirm and expand on these findings, but TAVR appears to be a safe option in patients with MAVD.

## 4. Conclusions

**Fig. 1.**
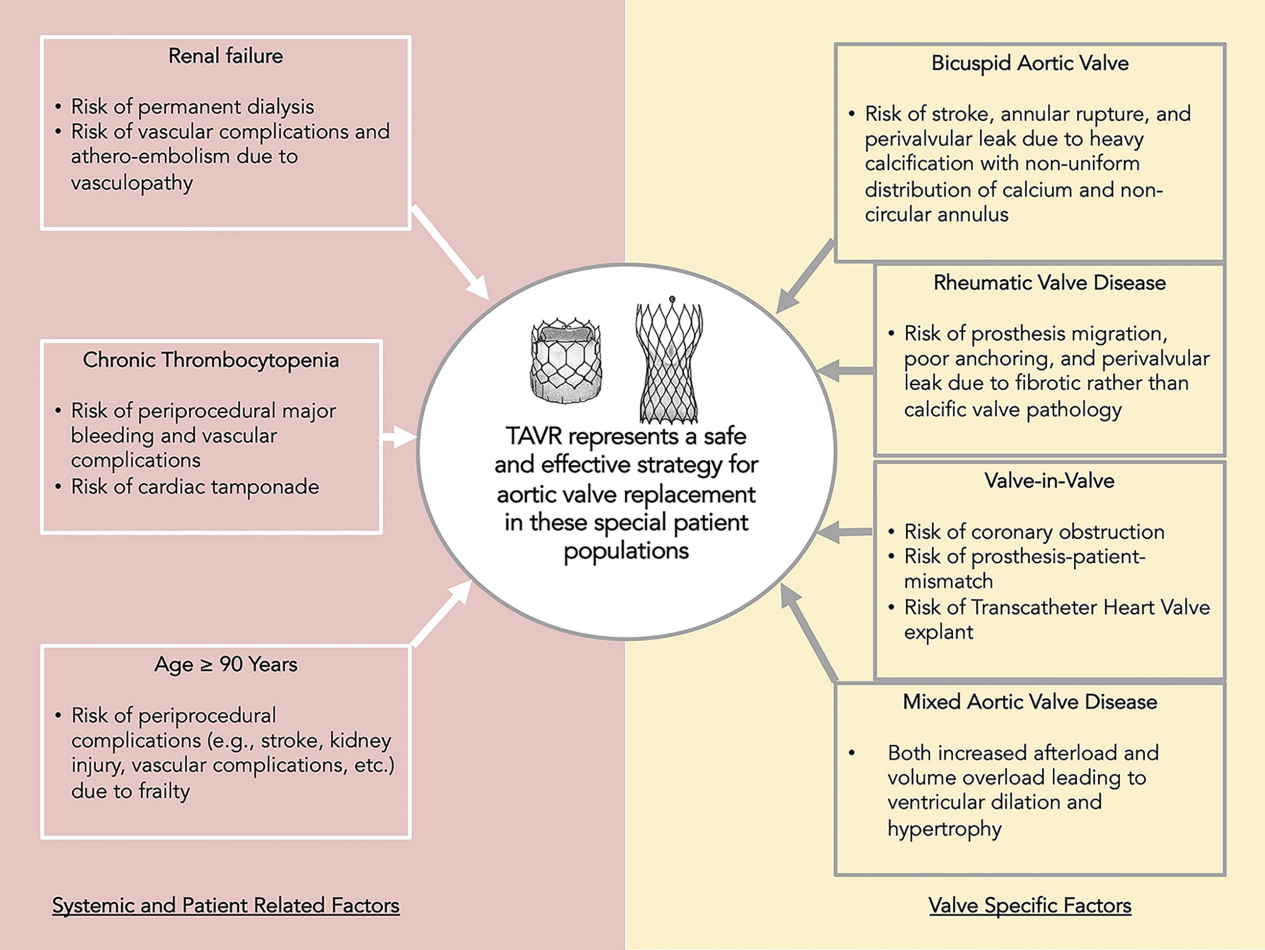
**Central Illustration**. Despite these considerations of each category of special population of patients, TAVR is an effective therapeutic strategy in severe aortic stenosis which offers improvement in quality of life as well as survival.

TAVR has effectively revolutionized the treatment of aortic stenosis. Since its inception and adoption, it has been shown to be a cost-effective solution improving both quality of life and mortality in patients across the entire range of surgical risks. Real world data has shown that it is also an effective strategy in patients with special risk conditions; both systemic and pertaining to valvular anatomy (Fig. 1). Further studies to optimize approach and improve outcomes in each of these higher risk conditions are imperative to advance the evolving therapy of TAVR for severe aortic stenosis.
